# The impact of the *MYB-NFIB* fusion proto-oncogene *in vivo*

**DOI:** 10.18632/oncotarget.9426

**Published:** 2016-05-18

**Authors:** Oliver R. Mikse, Jeremy H. Tchaicha, Esra A. Akbay, Liang Chen, Roderick T. Bronson, Peter S. Hammerman, Kwok-Kin Wong

**Affiliations:** ^1^ Department of Medicine, Dana Farber Cancer Institute, Boston, Massachusetts, USA; ^2^ Harvard Medical School, Boston, Massachusetts, USA; ^3^ Ludwig Institute for Cancer, Cambridge, Massachusetts, USA; ^4^ Department of Microbiology and Immunobiology, Division of Immunology, Harvard Medical School, Boston, Massachusetts, USA; ^5^ The Broad Institute, Cambridge, Massachusetts, USA; ^6^ Belfer Institute for Applied Cancer Science, Boston, Massachusetts, USA

**Keywords:** MYB-NFIB, adenoid cystic carcinoma, breast, salivary, GEMM

## Abstract

Recurrent fusion of the v-myb avian myelobastosis viral oncogene homolog (MYB) and nuclear factor I/B (NFIB) generates the MYB-NFIB transcription factor, which has been detected in a high percentage of individuals with adenoid cystic carcinoma (ACC). To understand the functional role of this fusion protein in carcinogenesis, we generated a conditional mutant transgenic mouse that expresses MYB-NFIB along with p53 mutation in tissues that give rise to ACC: mammary tissue, salivary glands, or systemically in the whole body. Expression of the oncogene in mammary tissue resulted in hyperplastic glands that developed into adenocarcinoma in 27.3% of animals. Systemic expression of the MYB-NFIB fusion caused more rapid development of this breast phenotype, but mice died due to abnormal proliferation in the glomerular compartment of the kidney, which led to development of glomerulonephritis. These findings suggest the MYB-NFIB fusion is oncogenic and treatments targeting this transcription factor may lead to therapeutic responses in ACC patients.

## INTRODUCTION

Adenoid cystic carcinoma (ACC) is a malignant tumor type that arises in the salivary gland, breast, respiratory airways, and vulva [1]–[2]. Salivary cancers constitute ~3%-6% of all head and neck neoplasms, and ACC is among the most common carcinomas of the salivary gland, affecting 10%-15% of patients [3]. In the salivary glands, ACC is a typically aggressive form of cancer that has poor long-term prognosis. About 80%-90% of patients with ACC in the head and neck die within 10-15 years after diagnosis [3] [4]. ACC in breast tissues constitutes ~1% of all invasive breast cancers and is categorized as triple-negative for estrogen receptor (ER), progesterone receptor (PR), and human epidermal growth factor receptor 2 (HER2). In contrast to salivary gland ACC, breast ACC has a non-aggressive phenotype and better prognosis [5]. Though extensively studied, very little remains known about the molecular pathogenesis of ACC.

Originally identified as a site for genomic integration for murine and avian retroviruses, accumulating evidence now suggests many roles for *v-myb avian myelobastosis viral oncogene homolog* (*MYB)* as a proto-oncogene. MYB functions primarily as a leucine zipper transcription factor that regulates expression of several families of genes, including housekeeping genes, cell differentiation factors, and oncogenes, including *MYC*, *BCL2,* and *HSP70* [6] [7]. Further, high MYB expression has been observed in proliferating endothelial, epithelial, and hematopoietic cells as well as leukemias, lymphomas, and tumors of the breast, colon, and pancreas [7] [8]. In addition, breast ACCs express high levels of *MYB* [5].

Reciprocal translocation between the terminal part of the long arm of chromosome 6 in *MYB* and the short arm of chromosome 9, within *nuclear factor I/B* (*NFIB)*, results in formation of a fusion gene that lacks its 3′-untranslated region, which contains target sequences for certain micro-RNAs. As with many such disruptions, this *MYB-NFIB* translocation leads to unusually high expression of *MYB* [8]. Recent studies have demonstrated a significant correlation between MYB-NFIB expression and ACC. MYB-NFIB expression** was present in 5 mammary ACCs and 6 head and neck ACCs analyzed in previous studies [4, 9, 10]. Another study performed whole exome sequencing using a combination of fusion transcript sequencing, quantitative real time PCR (RT-PCR), fluorescent in situ hybridization (FISH), and 3′ rapid amplification of cDNA ends and found the fusion was present in 19/24 cases of primary ACC (74%) [11].

Additionally, a more extensive study investigated the presence of *MYB-NFIB* fusion transcripts and *MYB* expression in more than 300 ACC tissues, including 75 salivary gland ACCs [12]. This study found fusion transcript mRNA in 28% of primary ACCs and 35% of metastatic ACCs. Further, an in-depth study of breast cancer samples using FISH and RT-PCR found the *MYB-NFIB* fusion gene in 12 out of 13 breast ACCs and mRNA expression in 4 out of 12 samples [5]. However, the remaining 8 samples did not produce RNA that was suitable to confirm the presence of the fusion transcript.

Given the compelling genomic and *in vitro* data implicating *MYB-NFIB* in ACC tumorigenesis, we further dissected the role of this novel fusion transcription factor *in vivo*. We investigated the role of *MYB-NFIB* in a novel genetically engineered mouse (GEM) model with overexpression in the salivary gland and breast compartments, either as a single genetic event or in conjunction with concurrent tumor suppressor *TP53* loss.

## RESULTS

### Characterization of MYB-NFIB expression in transgenic mice

We initially attempted to investigate the role of *MYB-NFIB* as a potential driver oncogene in salivary glands. MYB-NFIB mice were crossed with MMTV-Cre mice to obtain bi-allelic mice that expressed MYB-NFIB in salivary and breast tissues [15]. Additionally, as a more direct method of expressing MYB-NFIB in salivary tissue, we directly injected adenovirus-Cre into the salivary tissues of 6-week-old mice. Immunohistochemical examination of salivary tissue in both cohorts of mice showed low levels of expression of MYB, although tissues were otherwise normal with no evidence of malignancy even 12 months after induction (Figure S1).

### Characterization of breast tumors in MYB-NFIB transgenic mice

**Figure 1:**
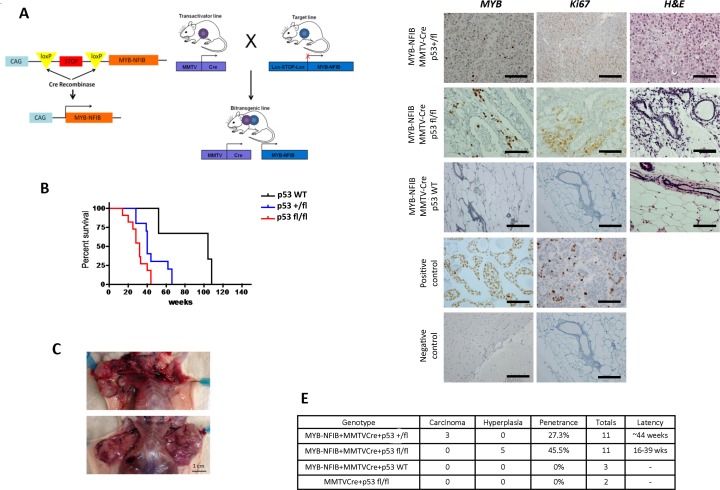
Activation of MYB-NFIB in conjunction with MMTV-Cre drives formation of breast adenocarcinoma in mice **A.** Schematic showing GEM model constitutively expressing the *MYB-NFIB* fusion crossed with MMTV-Cre mice to create MYB-NFIB/MMTV-Cre bi-transgenic mice. **B.** Kaplan-Meier survival curve of MYB-NFIB-expressing p53 homozygous wildtype (+/+), heterozygous (+/fl), or homozygous mutant (fl/fl) mice. **C.** Gross anatomy shows tumor burden of MYB-NFIB/MMTC-Cre/p53+/fl mice, with all tumors located in the upper mammary glands. Magnification bar = 1 cm. **D.** Representative H&E, MYB, and Ki67 staining images of poorly differentiated breast nodules. Magnification bars = 50 μm. **E.** Table summarizing different genotypic cohorts and corresponding tumor grade, penetrance, cohort size, and latency for mice that presented with mammary gland abnormalities.

Inactivation of *TP53* frequently occurs in ACC [5] [11]. One study identified a lack of p53 expression in 10 out of 13 breast ACC samples (76%) [5]. However, a recent study focusing on whole exome sequencing of 12 breast ACCs expressing *MYB-NFIB* fusion transcripts found a lack of mutations in *TP53* [16]. Therefore, the precise roles of MYB-NFIB and p53 in ACC remain to be determined. To address this question and accelerate tumor formation *in vivo,* we crossed MYB-NFIB mice with the p53fl/fl model to obtain tri-allelic mice and generated mice expressing wildtype, heterozygous mutant, or homozygous mutant p53 (Figure 1A). At 6 weeks of age, female tri-allelic mice were kept in breeding cages so they could experience multiple pregnancies, as this has been shown to be required for breast carcinoma development in MMTV-Cre models [15].

Introduction of the conditional p53 allele coincided with a reduction in average survival of mice—MYB-NFIB/MMTV-Cre/p53+/+ mice survived longest (~110 weeks), followed by MYB-NFIB/MMTV-Cre/p53+/fl mice (~65 weeks), and MYB-NFIB/MMTV-Cre/p53fl/fl mice had the shortest average lifespan (~42 weeks) (Figure 1B). MYB-NFIB/MMTV-Cre/p53+/+ mice did not develop tumors, while all mice in the p53+/fl cohort developed numerous tumors in upper mammary glands (Figure 1C). MYB-NFIB/MMTV-Cre/p53fl/fl mice presented hyperplastic breast phenotypes at the time of sacrifice due to progressive lymphoma.

To validate MYB-NFIB expression in the mouse model, immunohistochemistry was conducted on normal breast tissues and all tumor nodules acquired from the MMTV-Cre cohort. Although MYB staining was difficult to observe in normal breast tissue of the p53+/+ cohort, potentially due to lower cell density per section, p53+/fl and p53fl/fl mice showed specific nuclear staining for MYB (Figure 1D). Interestingly, p53+/fl mice displayed a higher breast tumor burden compared to p53fl/fl mice. Tumors varied in sizes, ranging from ~6-20 mm. Surprisingly, no mice in any of these cohorts presented salivary cancer.

Loss of p53 activity yielded an increased penetrance of breast cancer phenotypes and decreased latency—MYB-NFIB/MMTV-Cre/p53+/fl mice had penetrance of 27.3% and a latency of ~44 weeks, while MYB-NFIB/MMTV-Cre/p53fl/fl mice had penetrance of 45.5% and a latency of 16-39 weeks (Figure 1E). Control mice expressing MMTV-Cre/p53fl/fl alone did not present abnormal phenotypes in any analyzed tissues (data not shown).

**Figure 2:**
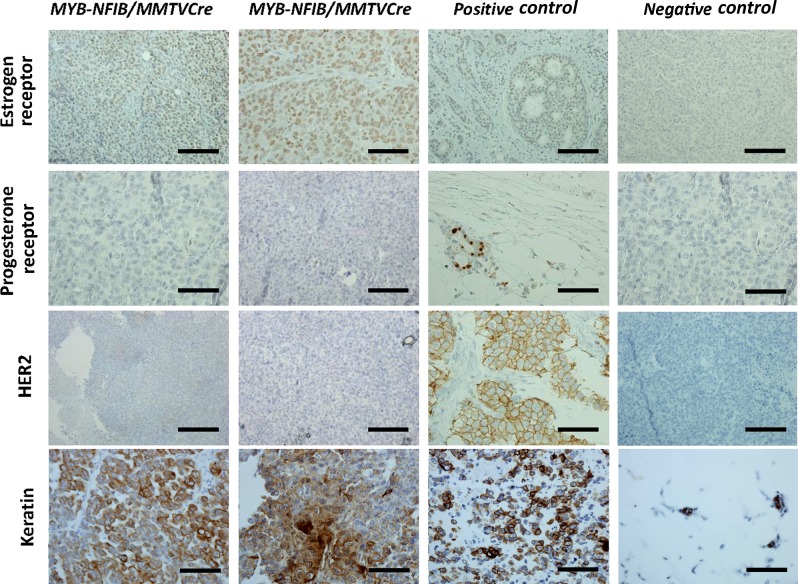
MYB-NFIB/MMTV-Cre/p53+/fl tumors express high levels of ER and keratin Representative images of immunohistochemical staining of ER, PR, HER2, and keratin in MYB-NFIB/MMTV-Cre/p53+/fl mice (2/3) and controls. Magnification bars = 50 μm.

A subset of MYB-NFIB/MMTV-Cre/p53+/fl mice presented tumors resembling poorly differentiated breast adenocarcinomas (Figure 1D, upper three panels). To further characterize the model, tumors were stained with keratin, Ki67, ER, PR, and HER2 antibodies (Figure 2). Tumors stained strongly for keratin, which is a known biomarker for ACC [1]. We also found increased Ki67 and ER staining, which coincides with findings that proliferation of ER-positive mammary cells have high levels of MYB [7] [15]. Statistical analysis correlating MYB and ER positivity showed no significant difference between the two datasets (Figure S2).

### Assessing MYB-NFIB expression on a global scale

Next, we conducted an additional study investigating the effects of widespread expression of this fusion gene. This was done to create an additional system with which we could investigate the role of MYB-NFIB expression in salivary tissue as well as other tissues. In addition, our study of MYB-NFIB using the MMTV-Cre driver system suggested a role for MYB-NFIB as an oncogenic driver of breast cancer. Therefore, we attempted to generate a model where we could study the role of MYB-NFIB as a driver of cancer in a more global setting.

**Figure 3:**
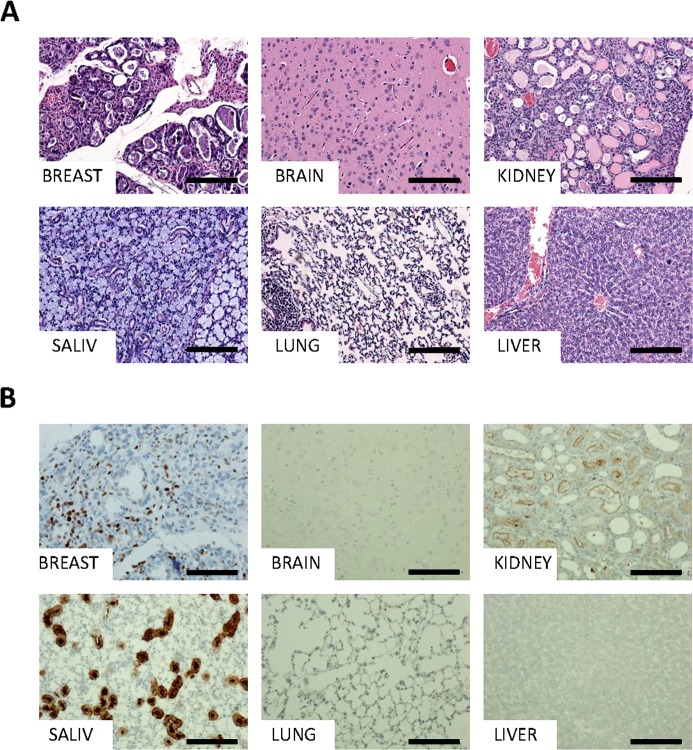
Detailed histology and characterization of MYB-NFIB mice Representative H&E **A.** and MYB immunohistochemical staining **B.** of MYB-NFIB/UCRE/p53fl/fl mouse breast, brain, kidney, salivary gland, lung, and liver tissues. Magnification bars = 50 μm.

To do this, MYB-NFIB/p53fl/fl mice were crossed to a tamoxifen-inducible Cre allele, UCRE, which resulted in widespread expression of MYB-NFIB in most tissues at varying levels. At 3 weeks post-induction, mice appeared emaciated and were sacrificed. Histological analysis of brain, breast, kidney, liver, lung, mammary gland, and salivary gland tissues revealed necrosis of the liver and abnormal kidney structures (Figure 3A). Closer examination of the kidneys revealed tubules that were dilated with albumin. Further, glomeruli appeared shrunken and atrophied, with no thickening of the basement membrane, and what appeared to be fusion of glomeruli to the Bowman's capsule (Figure 3A, upper right panel). In addition, this cohort displayed hyperplasia and aberrant lactation in breast tissues (Figure 3A, upper left panel). Kidney and liver abnormalities were detected in all mice in this cohort (*n* = 3), and the breast phenotype was detected in female mice exclusively (*n* = 2).

As with the MMTV-Cre cohort, immunohistochemical analysis of MYB-NFIB/UCRE/p53fl/fl tissues showed strong nuclear staining of MYB in breast tissues as well as kidney and several other tissues (Figure 3B). A control group of UCRE/p53fl/fl mice were continually dosed with tamoxifen for a period of six months and then sacrificed. This cohort showed no abnormalities in any tissues, and no MYB expression or increased Ki67 expression was evident through immunohistochemistry (Figure S3).

## DISCUSSION

Here we characterized development of the first MYB-NFIB-driven GEM model. The MMTV-Cre promoter has been extensively used in mouse models to drive gene expression in breast and salivary glands [15]. We applied this promoter to drive MYB-NFIB expression in salivary glands and mammary tissue to study tumorigenesis driven by the fusion gene. The dominant breast cancer phenotype of the model precluded study of *MYB-NFIB* as an oncogene in the salivary gland, despite observed expression in epithelial cells of this tissue. Although attempts to induce MYB-NFIB by direct injection in the salivary gland did not result in robust MYB-NFIB expression, our injection technique needs further optimization (Figure S1).

Although expression of MYB-NFIB has been shown in triple-negative (ER, PR, HER2) breast ACC [5], immunohistochemistry showed a strong correlation between expression of ER and MYB in higher grade tumors from our mouse model. Tumors also showed strong Ki67 and ER staining, which coincides with findings that proliferation of ER-positive cells in these tumors relies on MYB expression [5]. The incidence of ER-positivity has been shown to be as high as 70% across all breast cancers [15]. Therefore, our findings correlating expression of MYB-NFIB and ER in breast cancer could advance understanding and treatment potential of this specific category of breast cancer [15].

It should be noted that the relatively low number of mice that presented breast carcinoma (*n* = 3) and hyperplasia (*n* = 5) is a consequence of latency (as high as 44 weeks) and penetrance (as low as 23%) of the model presented in this study. In addition, although the MYB-NFIB/MMTV-Cre/p53+/fl cohort presented late stage carcinomas, which stained positive for keratin, our study does not show conclusively that these can be categorized as ACCs. Therefore, a more thorough study with larger cohorts would be ideal for future experiments involving extensive statistical, histologic, and expression analyses.

Additionally, MYB-NFIB/UCRE/p53fl/fl tri-allelic mice that globally express MYB-NFIB presented an unexpected phenotype of significantly reduced lifespan upon activation of *MYB-NFIB* at 8-9 weeks of age. Much to our surprise, upon analysis of all organs, these mice displayed necrotic livers and a kidney phenotype with severe accumulation of albumin and structural distortion reminiscent of glomerulonephritis. This is possibly due to capillary damage, which would result in shrunken capsids and an accumulation of albumin in the kidneys. In addition to this curious phenotype, female mice in this cohort also displayed hyperplasia in mammary glands. High expression of MYB-NFIB in kidney and breast tissues suggests *MYB-NFIB* as a possible driver of both phenotypes. Differences in phenotype latency in our two driver systems (MMTV-Cre *vs*. UCRE) is unknown, although differing expression levels may be an inherent consequence of the models.

In this study, we also attempted to address the contributions of p53 inactivation to the function of *MYB-NFIB* as an oncogenic driver. We found a correlation between increased tumorigenesis and loss of either one or both copies of *TP53*. We also detected increased fusion protein expression in the absence of functional p53, suggesting that MYB-NFIB may be unstable or degraded through unknown mechanisms in the presence of p53. Even though a recent study by Mitani et al. found a lack of *TP53* mutation in breast ACC, our results suggest that additional studies might be required to further elucidate the role of MYB and p53 in this context.

Previous studies have examined expression profiles of human breast ACCs, but these studies focused on comparisons between ACCs and ductal carcinomas of no special type [5]. To our knowledge, there have been no expression profile studies comparing late stage human breast ACC to early stage ACC or healthy breast tissue. Such a study would prove valuable to gain a better understanding of ACC development and its relation to MYB-NFIB.

Several potential therapeutic approaches have been proposed for targeting MYB in cancer. These include inhibition of ER itself or its ability to interact with MYB or targeting of MYB transcription targets [7]. In addition to its interaction with ER, MYB's interaction with coactivators, such as CBP/p300, is vital for its transactivation and transformation [7]. This interaction is also an avenue worth exploring for inhibitory agents. Direct targeting of MYB has also been considered through use of DNA vaccines that encode a fusion protein of two tetanus toxin peptides flanking the full-length *MYB* sequence [7] [8]. Finally, development of siRNA technology designed to target *MYB* provides a viable therapeutic opportunity [7].

In conclusion, these mouse studies demonstrate MYB-NFIB's potential as an oncogenic driver in the breast. Although we acknowledge limitations in our study in regards to latency, penetrance, and cohort size, as a novel GEM model our studies provide ample opportunities for further understanding the function of *MYB-NFIB* as an oncogenic driver and for the application of relevant therapeutics.

## MATERIALS AND METHODS

### Ethics statement

The Dana-Farber Institutional Animal Care and Use Committee (IACUC) reviewed and approved the animal procedures used in this study as documented by animal protocol 09-073. IACUC guidelines and regulations followed U.S. National Institute of Health guidelines, U.S. Public Health Service policy, and U.S. Food and Drug Administration regulations to minimize stress and pain on research animals. Monitoring health and wellbeing of animals was performed according to the Standard Operating Procedures of the Dana-Farber Cancer Institute's Animal Resource Facility, “Procedures for Reporting Animal Health Issues SOP T031.” This protocol designates a well-trained, attentive technician to assist in the detection of illness, distress, and disease through twice-a-day health checks. Any observed sign of injury, illness, disease, or distress was promptly reported to the veterinarian, clinical staff, and/or manager, who informed us of the designated animal with appropriate follow-up and treatment or euthanasia recommendations made by the veterinarian or technical services manager.

### Generation of MYB-NFIB transgenic mice

MYB-NFIB was generated from human cDNA using primers to amplify *MYB,* which was then modified to include the NFIB fusion at the 3′ end using PCR. The sequence was based on a prior study and used the human *MYB-NFIB* fusion variant 8 mRNA from the Genbank database (#NM_001161660.1) [4]. *MYB-NFIB* variant 8 was an ideal candidate because its sequence closely matched the predominant breakpoints found in this study—between *MYB* intron 14 and *NFIB* intron 8 [4] The complete sequence is available in Table S1. Sequence was verified and cloned into a modified transgenic vector and preceded by a lox-stop-lox sequence, which expressed *MYB-NFIB* upon exposure to Cre recombinase.

This construct was co-electroporated with a FLPe recombinase plasmid into v6.5 C57BL/6J (female) x 129/sv (male) embryonic stem cells (Open Biosystems) as described elsewhere [13] [14]. With this previously designed embryonic stem cell system, flip recombinase mediates recombination of the flip sites and integration of the transgene into the *ColA1* locus. Transgenic positive clones were selected for hygromycin resistance and evaluated for transgenic integration using PCR. Transgenic-positive stem cell clones were injected into black 6 blastocysts, and the resulting chimeras mated with BALB/c wildtype mice to confirm germline transmission of *MYB-NFIB* and expand experimental colonies.

### Breeding and tumor induction in transgenic mice

MYB-NFIB transgenic mice were bred with p53 conditional knockout mice (p53flox/flox, Jackson Laboratories #8462). MYB-NFIB mice were also bred with a strain that expresses Cre recombinase under the control of the mouse mammary tumor virus (MMTV) long terminal repeat promoter [B6129-Tg(MMTV-Cre)4Mam/J, Jackson Laboratories #003553] and a ubiquitin-Cre-ERT2 (UCRE) strain (Jackson Laboratories #007001). For the MYB-NFIB/UCRE cohort, 6-week-old mice were dosed with tamoxifen (5 mg/kg) three times a week for two weeks to activate Cre.

To introduce adenovirus-Cre into salivary tissues, 6-week-old MYB-NFIB mice were injected with adenovirus-Cre (5 × 10^7^ pfu, approximately 20 μL in DMEM; obtained from University of Iowa) directly into one side of the salivary tissue with a 27-gauge needle.

Mice that showed tumor progression up to a maximum of 2 cm in any dimension were culled for analysis. Mice showing rapid weight loss (≥15% of normal body weight), dyspnea, diarrhea, progressive dermatitis, or hair coat abnormalities prior to reaching the study endpoint were euthanized. Animal weight and onset of signs of distress were followed as described in the IACUC-approved protocol. Method of approved euthanasia for animals in our study was carbon dioxide asphyxiation on the basis of its rapidity, safety, and effectiveness in causing loss of brain function, followed by cervical dislocation. Unexpected deaths, although uncommon, did occur due to disease progression (most commonly occurring overnight), and all were appropriately reported.

### Immunohistochemistry and antibodies

Brain, salivary gland, lung, breast, liver, and kidney tissues were fixed in 10% buffered formalin overnight and then transferred to 70% ethanol. Lungs were inflated before fixation. Fixed tissues were embedded in paraffin and cut into thin sections. Sections were stained with the following antibodies: anti-v-Myb and c-Myb (Abcam #ab45150), anti-PR (Abcam #131486), anti-HER2/ErB2 (Cell Signaling #4290S), anti-alpha-ER and beta-ER (Biorbyt #orb10615), and anti-keratin (Abcam #6401). Staining kits for Ki67 (Vector #VP-K451) were performed per manufacturer's instructions. Figures show representative images for each cohort.

## SUPPLEMENTARY MATERIALS FIGURES AND TABLES


