# Supplementary material for: The impact of the MYB-NFIB fusion proto-oncogene in vivo
Source: Oncotarget. 2016 May 18;7(22):31681–8. doi: 10.18632/oncotarget.9426 (PMC5077968; doi:10.18632/oncotarget.9426)
Supplement: Supplementary file 1 [file oncotarget-07-31681-s001.pdf]

The impact of the *MYB-NFIB* fusion protooncogene *in vivo*

Supplementary Material

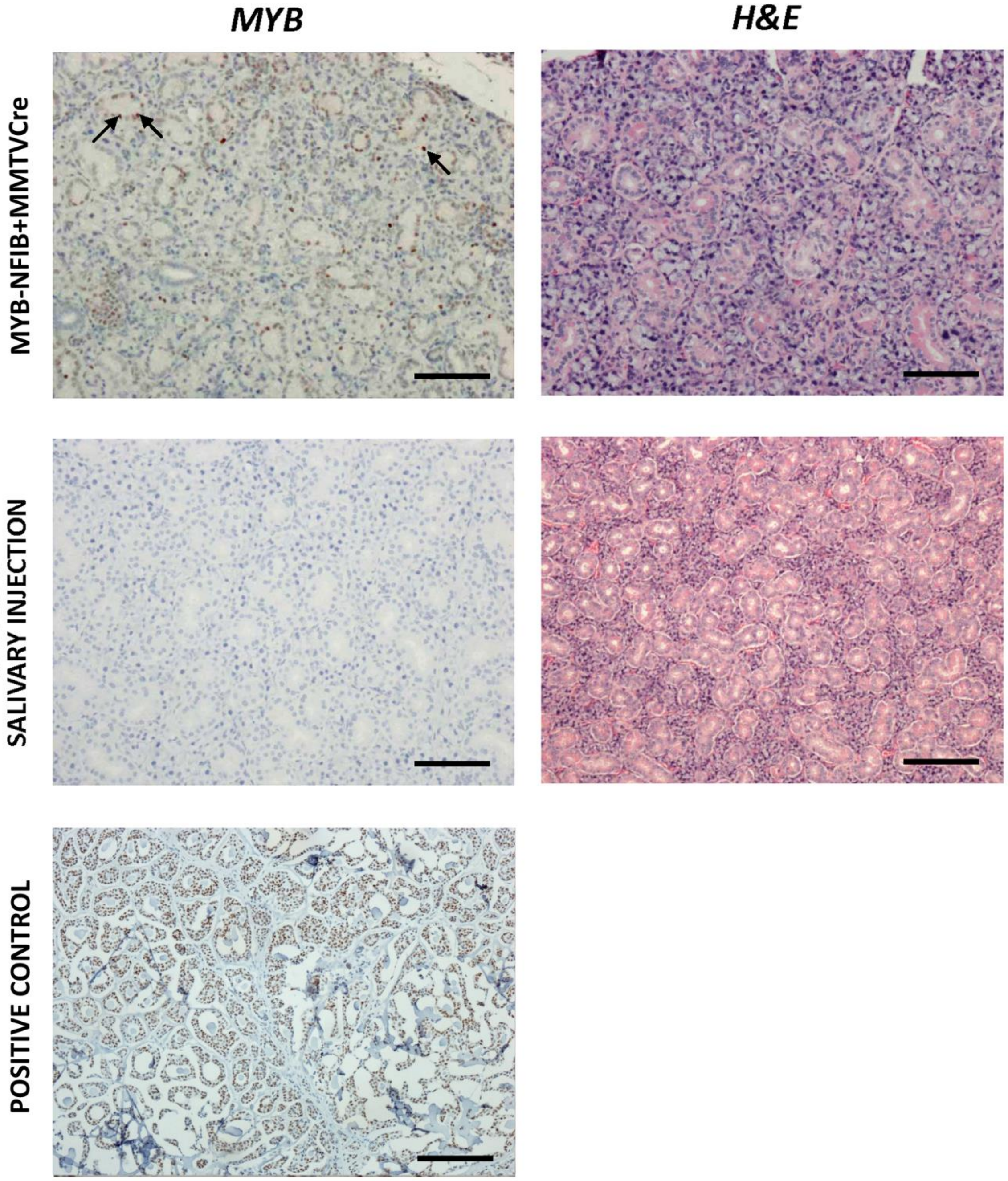

Figure S1: Expression of MYB-NFIB in salivary tissue confers lack of aberrant phenotype. H&E and MYB immunohistochemical staining of representative mice for each cohort. Expression of MYB-NFIB in salivary glands was induced through introduction of MMTV-Cre to create bi-transgenic mice (top two panels) or by injection of adenovirus-Cre (bottom two panels). MYB-NFIB/MMTV-Cre mouse shown is ~100 weeks old, and MYB-NFIB mouse shown is ~9 weeks post-injection. Arrows designate positive epithelial cells. Magnification bars = 50 μm.

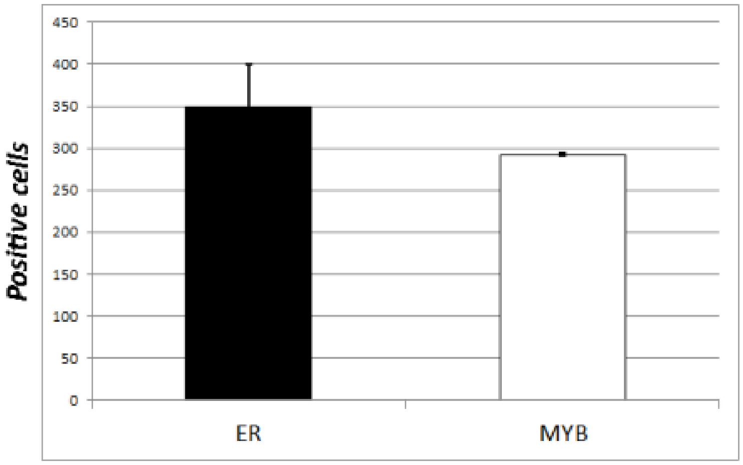

Figure S2: Expression of MYB and ER in breast tumors of MYB-NFIB/MMTV-Cre/p53+/fl mice. Random fields in immunohistochemically stained slides were captured at 40X magnification, and positive cells were counted in each panel. Student’s t-test showed no statistically significant difference between MYB- and ER-positive cells.

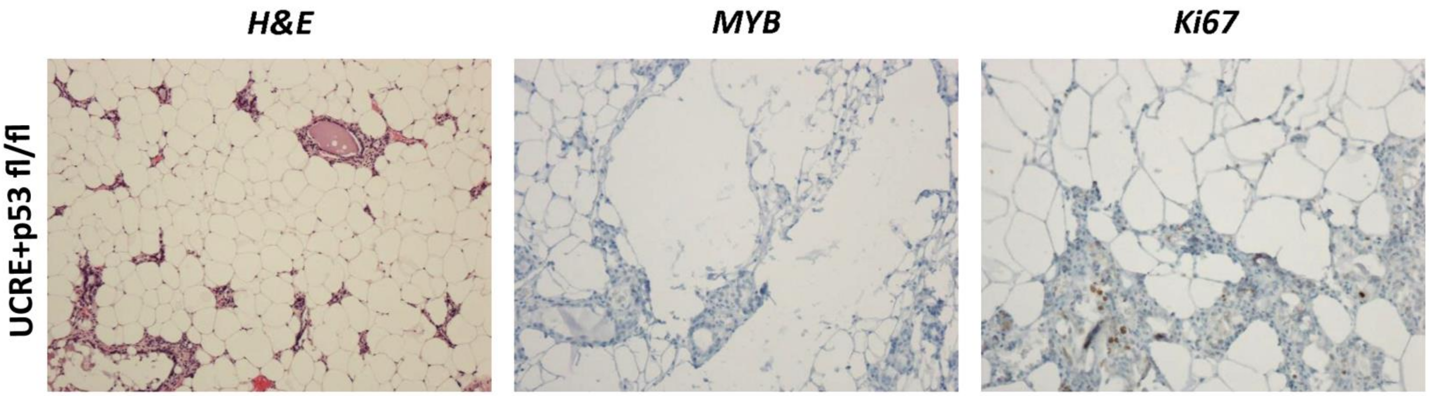

Figure S3: Lack of MYB-NFIB expression in UCRE/p53fl/fl mice confers lack of aberrant phenotype in mammary glands. H&E and immunohistochemical staining for MYB and Ki67 in mammary gland tissues of UCRE/p53fl/fl mice. Magnification bars = 50 μm.

Table S1: Complete sequence of MYB-NFIB fusion gene (Genbank NM_001161660.1)

Homo sapiens MYB proto-oncogene, transcription factor (MYB), transcript variant 8, mRNA

1	aatatcaacc	tgtttcctcc	tcctccttct	cctcctcctc	cgtgacctcc	tcctcctctt
61	tctcctgaga	aacttcgccc	cagcggtgcg	gagcgccgct	gcgcagccgg	ggagggacgc
121	aggcaggcgg	cgggcagcgg	gaggcggcag	cccggtgcgg	tccccgcggc	tctcggcgga
181	gccccgcgcc	cgccgcgcca	tggcccgaag	accccggcac	agcatatata	gcagtgacga
241	ggatgatgag	gactttgaga	tgtgtgacca	tgactatgat	gggctgcttc	ccaagtctgg
301	aaagcgtcac	ttggggaaaa	caaggtggac	ccgggaagag	gatgaaaaac	tgaagaagct
361	ggtggaacag	aatggaacag	atgactggaa	agttattgcc	aattatctcc	cgaatcgaac
421	agatgtgcag	tgccagcacc	gatggcagaa	agtactaaac	cctgagctca	tcaagggtcc
481	ttggaccaaa	gaagaagatc	agagagtgat	agagcttgta	cagaaatacg	gtccgaaacg
541	ttggtctgtt	attgccaagc	acttaaaggg	gagaattgga	aaacaatgta	gggagaggtg
601	gcataaccac	ttgaatccag	aagttaagaa	aacctcctgg	acagaagagg	aagacagaat
661	tatttaccag	gcacacaaga	gactggggaa	cagatgggca	gaaatcgcaa	agctactgcc
721	tggacgaact	gataatgcta	tcaagaacca	ctggaattct	acaatgcgtc	ggaaggtcga
781	acaggaaggt	tatctgcagg	agtcttcaaa	agccagccag	ccagcagtgg	ccacaagctt
841	ccagaagaac	agtcatttga	tgggttttgc	tcaggctccg	cctacagctc	aactccctgc
901	cactggccag	cccactgtta	acaacgacta	ttcctattac	cacatttctg	aagcacaaaa
961	tgtctccagt	catgttccat	accctgtagc	gttacatgta	aatatagtca	atgtccctca
1021	gccagctgcc	gcagccattc	agacacagaa	ccacacatgc	agctaccccg	ggtggcacag
1081	caccaccatt	gccgaccaca	ccagacctca	tggagacagt	gcacctgttt	cctgtttggg
1141	agaacaccac	tccactccat	ctctgccagc	ggatcctggc	tccctacctg	aagaaagcgc
1201	ctcgccagca	aggtgcatga	tcgtccacca	gggcaccatt	ctggataatg	ttaagaacct
1261	cttagaattt	gcagaaacac	tccaatttat	agattctttc	ttaaacactt	ccagtaacca
1321	tgaaaactca	gacttggaaa	tgccttcttt	aacttccacc	cccctcattg	gtcacaaatt
1381	gactgttaca	acaccatttc	atagagacca	gactgtgaaa	actcaaaagg	aaaatactgt
1441	tttagaacc	ccagctatca	aaaggtcaat	cttagaaagc	tctccaagaa	ctcctacacc
1501	attcaaacat	gcacttgcag	ctcaagaaat	taaatacggt	cccctgaaga	tgctacctca
1561	gacaccctct	catctagtag	aagatctgca	ggatgtgatc	aaacaggaat	ctgatgaatc
1621	tggaattgtt	gctgagtttc	aagaaaatgg	accaccctta	ctgaagaaaa	tcaaacaaga
1681	ggtggaatct	ccaactgata	aatcaggaaa	cttcttctgc	tcacaccact	gggaagggga
1741	cagtctgaat	acccaactgt	tcacgcagac	ctcgcctgtg	gcagatgcac	cgaatattct
1801	tacaagctcc	gttttaatgg	caccagcatc	agaagatgaa	gacaatgttc	tcaaagcatt
1861	tacagtacct	aaaaacaggt	ccctggcgag	cccccttgcag	ccttgtagca	gtacctggga
1921	acctgcatcc	tgtggaaaga	tggaggagca	gatgacatct	tccagtcaag	ctcgtaaata
1981	cgtgaatgca	ttctcagccc	ggacgctggt	catgtgagac	atttccagaa	aagcattatg
2041	gttttcagaa	cacttcaagt	tgacttggga	tatatcattc	ctcaacatga	aacttttcat
2101	gaatgggaga	agaacctatt	tttgttgtgg	tacaacagtt	gagagcagca	ccaagtgcat
2161	ttagttgaat	gaagtcttct	tggatttcac	ccaactaaaa	ggatttttaa	aaataaataa
2221	cagtcttacc	taaattatta	ggtaatgaat	tgtagccagt	tgttaatatc	ttaatgcaga
2281	tttttttaaa	aaaaacataa	aatgatttat	ctgtatttta	aaggatccaa	cagatcagta
2341	tttttttcctg	tgatgggttt	tttgaaattt	gacacattaa	aaggtactcc	agtatttcac
2401	ttttctcgat	cactaaacat	atgcatatat	ttttaaaaat	cagtaaaagc	attactctaa
2461	gtgtagactt	aataccatgt	gacatttaat	ccagattgta	aatgctcatt	tatggttaat
2521	gacattgaag	gtacatttat	tgtaccaaac	cattttatga	gttttctgtt	agcttgcttt
2581	aaaaattatt	actgtaagaa	atagttttat	aaaaaattat	atttttattc	agtaatttaa
2641	ttttgtaaat	gccaaatgaa	aaacgttttt	tgctgctatg	gtcttagcct	gtagacatgc
2701	tgctagtatc	agaggggcag	tagagcttgg	acagaaagaa	aagaaacttg	gtgttaggta
2761	attgactatg	cactagtatt	tcagactttt	taattttata	tatatataca	tttttttttcc
2821	ttctgcaata	catttgaaaa	cttgtttggg	agactctgca	tttttattg	tggttttttt
2881	gttattgttg	gtttatacaa	gcatgcgttg	cacttctttt	ttgggagatg	tgtgttgttg
2941	atgttctatg	ttttgttttg	agtgtagcct	gactgtttta	taatttggga	gttctgcatt
3001	tgatccgcat	cccctgtggt	ttctaagtgt	atggtctcag	aactgttgca	tggatcctgt
3061	gtttgcaact	ggggagacag	aaactgtggt	tgatagccag	tcactgcctt	aagaacattt
3121	gatgcaagat	ggccagcact	gaacttttga	gatatgacgg	tgtacttact	gccttgtagc
3181	aaaataaaga	tgtgccctta	ttttacctac			
